# ChemMaps: Towards an approach for visualizing the chemical space based on adaptive satellite compounds

**DOI:** 10.12688/f1000research.12095.2

**Published:** 2017-08-04

**Authors:** J. Jesús Naveja, José L. Medina-Franco

**Affiliations:** 1 Department of Pharmacy, School of Chemistry, Universidad Nacional Autónoma de México, Mexico City, 04510, Mexico; 2 PECEM, Faculty of Medicine, Universidad Nacional Autónoma de México, Mexico City, 04510, Mexico

**Keywords:** chemical space, data visualization, epigenetics, principal components analysis, similarity matrix

## Abstract

We present a novel approach called ChemMaps for visualizing chemical space based on the similarity matrix of compound datasets generated with molecular fingerprints’ similarity. The method uses a ‘satellites’ approach, where satellites are, in principle, molecules whose similarity to the rest of the molecules in the database provides sufficient information for generating a visualization of the chemical space. Such an approach could help make chemical space visualizations more efficient. We hereby describe a proof-of-principle application of the method to various databases that have different diversity measures. Unsurprisingly, we found the method works better with databases that have low 2D diversity. 3D diversity played a secondary role, although it seems to be more relevant as 2D diversity increases. For less diverse datasets, taking as few as 25% satellites seems to be sufficient for a fair depiction of the chemical space. We propose to iteratively increase the satellites number by a factor of 5% relative to the whole database, and stop when the new and the prior chemical space correlate highly. This Research Note represents a first exploratory step, prior to the full application of this method for several datasets.

## Introduction

Visual representation of chemical space has multiple implications in drug discovery for virtual screening, library design and comparison of compound collections, among others ^1^. Amongst the multiple methods to explore chemical space, principal component analysis (PCA) of pairwise similarity matrices computed with structural fingerprints has been used to analyze compound datasets ^2, 3^. A drawback of this approach is that it becomes impractical for large libraries due to the large dimension of the similarity matrix ^4^. Other approaches use molecular representations different from structural fingerprints, such as physicochemical properties or complexity descriptors, or methods different from PCA, such as multidimensional-scaling and neural networks ^5, 6^.

In representation of the chemical space based on PCA there have been “chemical satellite” approaches, such as ChemGPS, which select satellites molecules that might not be included in the database to visualize, but have extreme features that place them as outliers, with the intention to reach as much of the chemical space as possible ^7– 10^. Also, a related and more recent approach, Similarity Mapplet, makes possible the visualization of very large chemical libraries, by considering PCA of different molecular features, including structural ^11^.

Although we concur with the fact that not all compounds in a compound data set should be necessary to generate a meaningful chemical space, there are still obvious limitations of using a fixed set of satellites to which the user is blinded. Also, until now there was no proposal of such a method based on structural similarity.

We therefore suggest the hybrid approach, ChemMaps, in which a portion of the database to be represented is used as satellite, thereby decreasing the computational effort required to compute the similarity matrix without losing adaptability of the method to any particular database. Since it is expected that more diverse sets would require more satellites, a second goal of this study was to qualitatively explore the relationship between the internal diversity of compound datasets and the fraction of compounds required as satellites, in order to generate a good approximation of the chemical space.

## Methods

Table 1 summarizes the six compound data sets considered in this study. Note that small median similarity values imply higher diversity. The datasets were selected from a large scale study of profiling epigenetic datasets (unpublished study, Naveja JJ and Medina-Franco JL) with relevance in epigenetic-drug discovery. We also included DrugBank as a control diverse dataset ^12^. Briefly, we selected focused libraries of inhibitors of DNMT1 (a DNA-methyltransferase; library diverse 2D and 3D), L3MBTL3 (a histone methylation reader; diverse 3D and less diverse 2D), SMARCA2 (a chromatin remodeller; diverse 2D, less diverse 3D), and CREBBP (a histone acetyltransferase; less diverse both 2D and 3D). Datasets were selected based on their different internal diversity (as measured with Tanimoto index/MACCS keys for 2D measurements and Tanimoto combo/OMEGA-ROCS for 3D; see Figure S1 in Supplementary File 1). Data sets in this work have approximately the same number of compounds except for HDAC1 and DrugBank, which were selected to benchmark the method in larger databases ( Table 2). We evaluated 2D diversity using the median of Tanimoto/MACCS similarity measures in KNIME version 3.3.2, and 3D diversity using the median of Combo Score from the ROCS, version 3.2.2 and OMEGA, version 2.5.1, OpenEye software ^13– 16^.

**Table 1.** Compound data sets used in the study.

Dataset	Description	Size	2D similarity ^a^	2D similarity ^b^	3D similarity ^c^
DNMT1 inhibitors	DNA-methyltransferase	244	0.44	0.12	0.16
SMARCA2 inhibitors	Chromatin remodeller	220	0.51	0.15	0.23
CREBBP inhibitors	Histone acetyltransferase	178	0.67	0.22	0.16
L3MBTL3 inhibitors	Histone methylation reader	115	0.77	0.41	0.03
HDAC1 inhibitors	Histone acetyltransferase	3,257	0.49	0.16	0.12
DrugBank	Approved drugs	1,900	0.35	NC	NC

^a^Median of Tanimoto/MACCS similarity; ^b^Median of Tanimoto/ECFP4 similarity; ^c^Median of OMEGA-ROCS similarity; NC: not calculated

To assess the hypothesis of this work we performed two main approaches A): *Backwards approach*: start with computing the full similarity matrix of each data set and remove compounds systematically; and B) *Forward approach:* start adding compounds to the similarity matrix until finding the reduced number of required compounds (called ‘satellites’) to reach a visualization of the chemical space that is very similar to computing the full similarity matrix. The second approach would be the usual and realistic approach from a user standpoint. Each method is further detailed in the next two subsections.

### Backwards approach

The following steps were implemented in an automated workflow in KNIME, version 3.3.2 ^17^:

1. For each compound in the dataset with *N* compounds, generate the *N* X *N* similarity matrix using Tanimoto/extended connectivity fingerprints radius 4 (ECFP4) generated with CDK KNIME nodes.

2. Perform PCA of the similarity matrix generated in step 1 and selected the first 2 or 3 principal components (PCs).

3. Compute all pair-wise Euclidean distances based on the scores of the 2 or 3 PCs generated in step 2. The set of distances are later used as reference or ‘ *gold standard*’. It should be noted that the “real” distances or true gold standard would consider the whole distance matrix. However, for visualization purposes it is unfeasible to render more than 3 dimensions. Therefore, we selected as reference the best 2D or 3D visualization possible by means of PCA.

4. Repeat steps 1 to 3 with one compound as satellite, generating an *N X 1* similarity matrix. The first compound was selected randomly. In this case, for example, it is only possible to calculate one PC, but as the number of satellites increases, we can again compute 2 or 3 PCs.

5. Calculate the correlation among the pairwise distances generated in step 2 obtained using the whole matrix (e.g., *gold standard*) and those obtained in step 4.

6. Iterate over steps 4 and 5 increasing the number of satellites one by one until *N - 1* satellites are reached. To select the second, third, etc. compounds, two approaches were followed: select compounds at random and select compounds with the largest diversity to the previously selected (i.e., Max-Min approach).

7. Estimate the proportion of satellite compounds required to preserve a ‘high’ (of at least 0.9) correlation.

8. The prior steps were repeated five times for each dataset in order to capture the stability of the method.

### Forward approach

The former approach is useful only for validation purposes of the methodology as a proof-of-principle. However, the obvious objective of a satellite-approach is to avoid the calculation of the complete similarity matrix e.g., step 1 in backwards approach. To this end, we developed a satellite-adding or forward approach, in contrast with the formerly introduced backwards approach. We started with 25% of the database as satellites and for each iteration we added 5% until the correlation of the pairwise Euclidean distances remains high (at least 0.9). A further description of the methods for standardizing the chemical data and integrating the dataset can be found in the Supplementary material, as well as a further description of the PCA analysis used.

This file contains the six compound datasets used in this work in SDF format No special software is required to open the SDF files. Any commercial or free software capable of reading SDF files will open the data sets supplied. Click here for additional data file. Copyright: © 2017 Naveja JJ and Medina-Franco JL 2017 Data associated with the article are available under the terms of the Creative Commons Zero "No rights reserved" data waiver (CC0 1.0 Public domain dedication).

## Results

### Backwards approach

**Figure 1.**
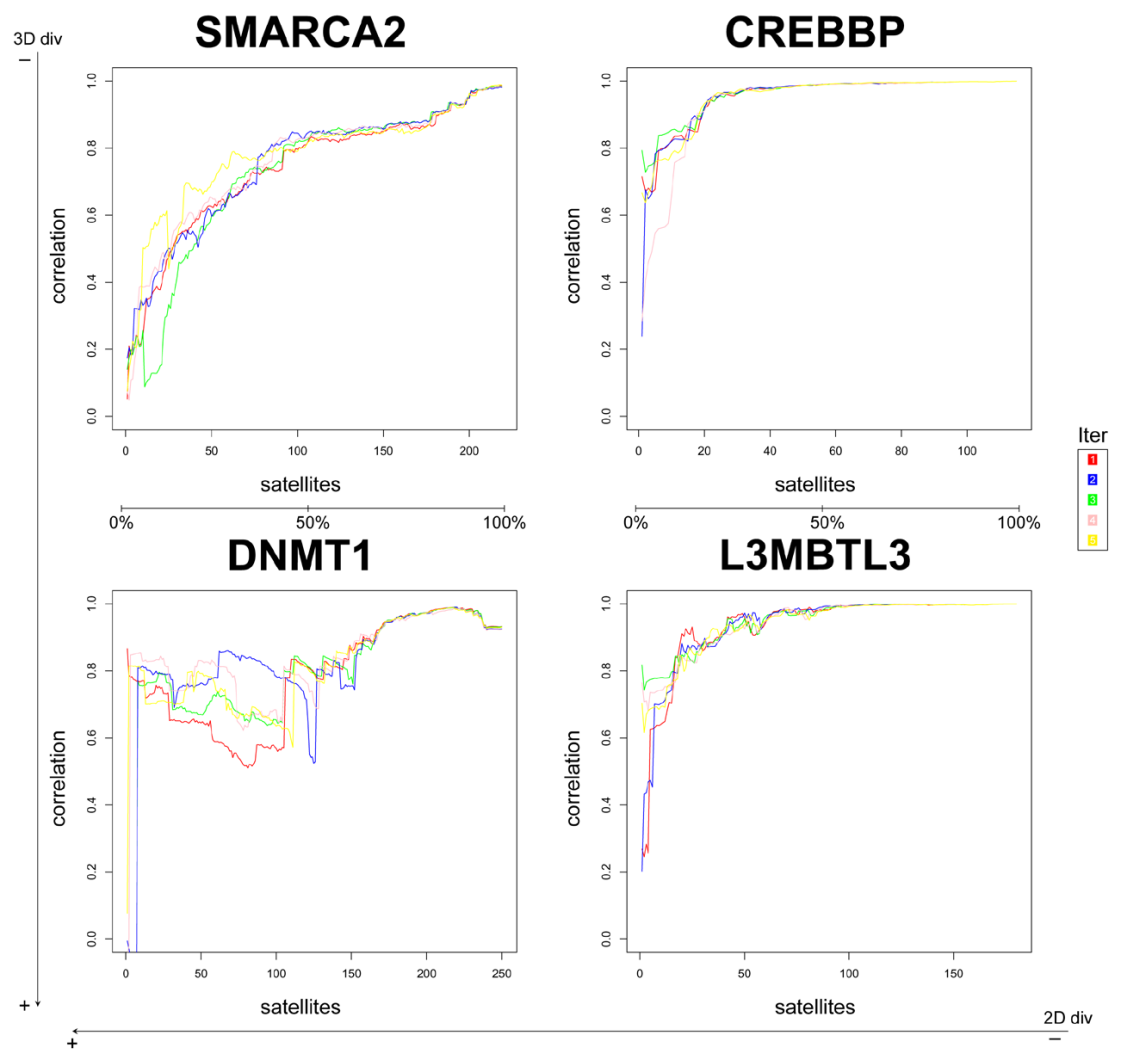
Backwards analysis with 2PCs picking satellites by diversity. The correlation with the results from the whole matrix was calculated with increasing numbers of satellites. Each colored line represents one of the five iterations.

**Figure 2.**
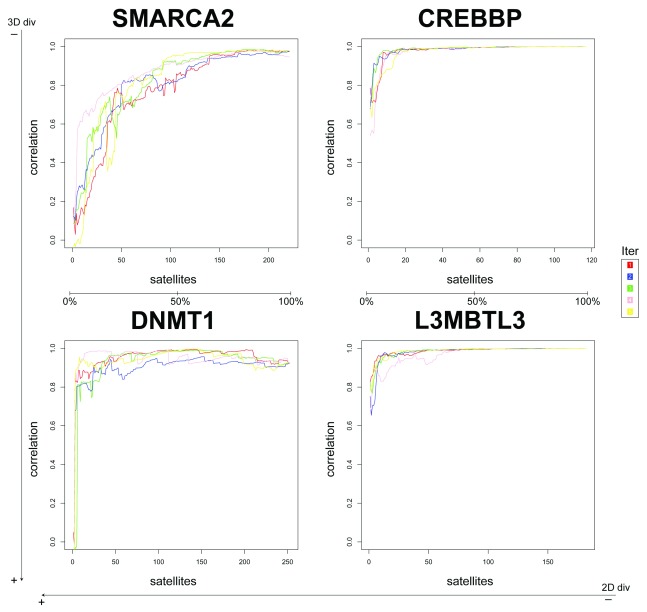
Backwards analysis with 2PCs picking satellites at random. The correlation with the results from the whole matrix was calculated with increasing numbers of satellites. Each colored line represents one of the five iterations.

In this pilot study, we assessed a few variables to tune up the method, such as the number of PCs used (2 or 3) and the selection of satellites at random or by diversity. We found that selection at random is more stable, above all in less diverse datasets ( Figure 1 and Figure 2; Figure S2 and Figure S3). Likewise, selecting 2 PCs the performance is slightly better and more stable (compare Figure 1 and Figure 2 against Figure S2 and Figure S3).

Therefore, from this point onwards we will focus on the results of the at random satellites selection and using 2 PCs ( Figure 2). From the four datasets, we conclude that for datasets with lower 2D diversity (CREBBP and L3MBTL3, see Table 1), around 25% of satellite compounds are enough to obtain a high correlation (≥ 0.9) with the gold standard (e.g., PCA on the whole matrix), *w*hereas for 2D-diverse datasets i.e., DNMT1 and SMARCA2, up to 75% of the compounds could be needed to ensure a high correlation. Nonetheless, even for these datasets, using 25% of the compounds as satellites the correlation with the gold standard is already between 0.6 and 0.8; using 50% of the compounds as satellites the correlation is between 0.7 and 0.9. Hence, the higher the diversity of a dataset (especially 2D), the higher the number of satellites required.

### Forward approach

**Figure 3.**
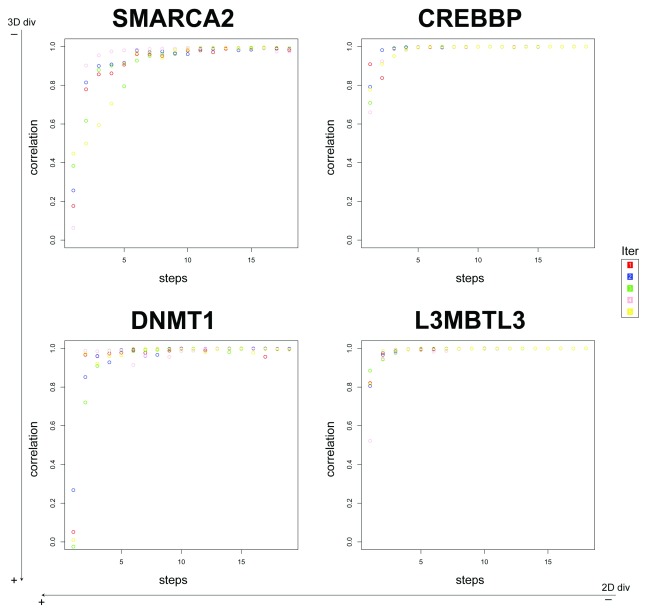
Forward analysis with 2PCs picking satellites at random step sizes of 5%.

Evidently, a useful method for reducing computing time and disk space usage should not use the PCA on the whole similarity matrix to determine an adequate number of satellites for each dataset. With that in mind, we decided to design a method that starts with a given percentage of the database as satellites, and then keeps adding a proportion of them until the correlation between the former and the updated data is of at least 0.9. In Figure 3 we depict this approach on the same databases in Table 1 for step sizes of 5% and starting from zero. Similarly as what we saw in the backwards method, around 5 steps (25% of the database) are usually necessary to reach a stable, high correlation between steps. Figure S4 shows that for step sizes of 10% there is no further improvement. Therefore we suggest that the method should, for default, start with 25% of compounds as satellites and then keep adding 5% until a correlation between steps of at least 0.9 is reached.

### Application

**Figure 4.**
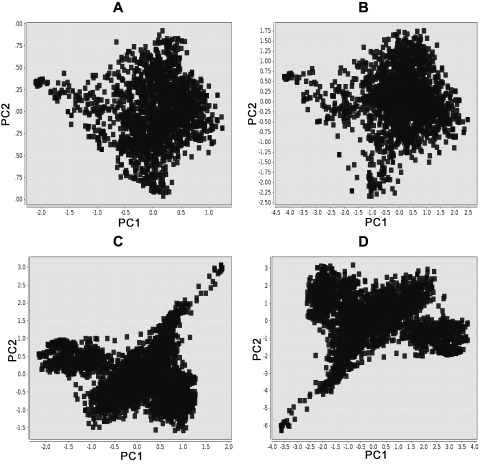
Chemical space of DrugBank using ( **A**) the adaptive satellites approach or ( **B**) the gold standard. As well as for HDAC1 using ( **C**) the adaptive satellites approach or ( **D**) the gold standard.

In this pilot study we applied the ChemMaps method to visualize the chemical space of two larger datasets (HDAC1 and DrugBank with 3,257 and 1,900 compounds, respectively, Table 1). As shown in Table 2, a significant reduction in time performance was achieved as compared to the gold standard, and the correlation between the gold standard and the satellites approach was in both cases higher than 0.9. Figure 4 depicts the chemical spaces generated in both instances. Although the orientation of the map changed for HDAC1, the shape and distances remain quite similar, which is the main objective. This preliminary work supports the hypothesis that a reduced number of compounds is sufficient to generate a visual representation of the chemical space (based on PCA of the similarity matrix) that is quite similar to the chemical space of the PCA of the full similarity matrix.

**Table 2.** Benchmark with larger databases.

Database	Gold standard timing (s)	Satellites timing (s)	Correlation
DrugBank	162	147	0.92
HDAC1	406	287	0.99

## Conclusion and future directions

This proof-of-concept study suggests that using the adaptive satellite compounds ChemMaps is a plausible approach to generate a reliable visual representation of the chemical space based on PCA of similarity matrices. The approach works better for relatively less-diverse datasets, although it seems to remain robust when applied to more diverse datasets. For datasets with small diversity, fewer satellites seem to be enough to produce a representative visual representation of the chemical space. The higher relevance of 2D diversity over 3D in this study could be importantly related to the fact that the chemical space depiction is based on 2D fingerprints. Therefore, the performance of the methods depicting the chemical space based on 3D fingerprints could also be assessed.

A major next step is to conduct a full benchmark study to assess the general applicability of the approach proposed herein, and also in larger databases, in which we anticipate this method would be even more useful. A second step is to propose a metric that determines the number of compounds required as satellites for PCA representation of the chemical space based on similarity matrices. As well, it is pending the development of quantitative metrics for assessing the stability of the satellites selection and thus conclusively establish the superiority of at random satellite selection. Finally, a more comprehensive and in-depth study of this new methodology should be addressed, in order to further characterise its applicability domain, including a dataset diversity threshold above which the confiability of the approach decreases.

## Data availability

The data referenced by this article are under copyright with the following copyright statement: Copyright: © 2017 Naveja JJ and Medina-Franco JL

Data associated with the article are available under the terms of the Creative Commons Zero "No rights reserved" data waiver (CC0 1.0 Public domain dedication). 

**Dataset 1. This file contains the six compound datasets used in this work in SDF format.** No special software is required to open the SDF files. Any commercial or free software capable of reading SDF files will open the data sets supplied. http://dx.doi.org/10.5256/f1000research.12095.d171632 ^18^

## Supplementary material

**Supplementary File 1: File with supporting methods, results and five figures.** Figure S1: 3D-Consensus Diversity Plot depicting the diversity of the datasets used for the backwards approach; Figure S2: Backwards analysis with 3PCs picking satellites by diversity; Figure S3: Backwards analysis with 3PCs picking satellites at random; Figure S4: Forward analysis with 2PCs picking satellites at random with step sizes of 10%; Figure S5: Plot of the percentage of variance explained by each principal component in the studied datasets.
