# Supplementary material for: ChemMaps: Towards an approach for visualizing the chemical space based on adaptive satellite compounds
Source: F1000Res. 2017 Aug 4;6:Chem Inf Sci-1134. Originally published 2017 Jul 17. [Version 2] doi: 10.12688/f1000research.12095.2 (PMC5538041; doi:10.12688/f1000research.12095.2)
Supplement: Supplementary file 2 [file f1000research-6-13342-s0001.tgz › b042f77d-79fa-44a6-a392-b37fb42c9df1.pdf]

Supplementary Material

ChemMaps: Towards an Approach for Visualizing the Chemical Space Based on Adaptative Satellite Compounds

J. Jesús Naveja [a,b] and José L. Medina-Franco [a]

<u>**Contents**</u> <u>Page</u>

**Supplementary Methods**	S2
**Supplementary Results**	S2
**Supplementary Table S1.** Correlation matrices of the Euclidean distances calculated with the whole matrix, 3PCs and 2PCs.	S3
**Supplementary references**	S4
**Figure S1**. 3D-Consensus Diversity Plot depicting the diversity of the datasets used for the backwards approach	S6
**Figure S2**. Backwards analysis with 3PCs picking satellites by diversity	S7
**Figure S3**. Backwards analysis with 3PCs picking satellites at random	S8
**Figure S4.** Forward analysis with 2PCs picking satellites at random with step sizes of 10%	S9
**Figure S5.** Plot of the percentage of variance explained by each principal component in the studied datasets	S10

Supplementary Methods

Databases

We combined structure-activity data from ChEMBL 22.1 [1], DrugBank 5.0.2 [2], PDSP Ki Database [3], LigandExpo 1 [4], Binding DB [5], T3DB [6] and the TTD 4.3.02 [7]. Prior molecule preprocessing and standardization using MOE, CDK and RDKit nodes in KNIME, compounds were clustered using INCHI keys, as well as DrugBank and ChEMBL IDs when available. Compound names were mapped to DrugBank synonyms, and chemical structures were converted to INCHI codes as calculated in KNIME 3.3 with RDKit nodes. If molecules were too similar (i.e., INCHI codes matched perfectly, or they had Tanimoto coefficient > 0.7 using ECFP4 and target similarity > 0.7), they were clustered as well. We removed PAINS compounds using a published KNIME workflow [8].

Target-compound associations were sometimes repeated within and among databases, sometimes with different potency data. All associations originated from qualitative datasets (i.e., DrugBank, LigandExpo, T3DB and TTD) were regarded as active. Quantitative datasets required further curation. For them, we considered a threshold of 5 in terms of logarithmic potency data (e.g., pIC_{50}, $pKi_{50} = 5$) for considering a compound to be “active”. If more than one measurement was available, in order to improve consistency of the data, we removed associations with many contradictory results. We thereby obtained data for 52 epigenetic targets, that will be submitted for publication in short. We selected 4 datasets with similar number of compounds (~200) and different 2D and 3D library diversities.

Molecular descriptors

Using KNIME 3.3 [9] RDKit and CDK nodes, we calculated ECFP4 (1024-bits) and MACCS keys (166-bits) fingerprints for all the structures. For assessing tridimensional database diversity, we computed 3D similarity of maximum 10 conformers (for lessening the computational expenditure) with ROCS version 3.2.1.4 algorithm implemented in OpenEye [10,11]. The conformer libraries were generated with OMEGA, version 2.5.1.4 from OpenEye [12,13].

Supplementary Results

Principal components analysis

Given that we were designing a method that would be more efficient than the best available 2D or 3D chemical spaced representation through PCA of the similarity matrix, we used the first 2 or 3PCs Euclidean distance using all compounds as satellites (i.e., the whole similarity matrix). Supplementary Figure 5 presents a plot of the percentage of variances explained by each of the principal components. For more diverse libraries, subsequent principal components are more relevant (i.e., in SMARCA2, HDAC1 and DrugBank, although not for DNMT1).

We compared our “operative” gold standard (using 2 or 3 PCs), against the whole matrix (using all principal components) by means of the correlation of the pairwise Euclidean distances, as shown in Supp Table 1. Interestingly, there is a high correlation (>0.9) in most of the smaller datasets (except SMARCA2, which is 2D diverse, and still retains a correlation >0.7), but this pattern is disrupted in HDAC1 and DrugBank datasets (correlations between 0.4 and 0.5). This is, however, a point against the use of PCA in general, and does not directly affect the applicability of the ChemMaps approach as an approximation of the best possible visualization through PCA.

Supplementary Table 1. Correlation matrices of the Euclidean distances calculated with the whole matrix, 3PCs and 2PCs. Interestingly, for larger libraries, the correlations drop drastically.

**Dataset**		*Whole matrix*	*3PCs*	*2PCs*
**L3MBTL3**	*Whole matrix*	-	-	-
	3PCs	0.992	-	-
	2PCs	0.983	0.986	-
**CREBBP**	*Whole matrix*	-	-	-
	3PCs	0.988	-	-
	2PCs	0.977	0.987	-
**SMARCA2**	*Whole matrix*	-	-	-
	3PCs	0.785	-	-
	2PCs	0.766	0.918	-
**DNMT1**	*Whole matrix*	-	-	-
	3PCs	0.923	-	-
	2PCs	0.913	0.966	-
**HDAC1**	*Whole matrix*	-	-	-
	3PCs	0.420	-	-
	2PCs	0.421	0.989	-
**DrugBank**	*Whole matrix*	-	-	-
	3PCs	0.449	-	-
	2PCs	0.441	0.994	-

Supplementary References

1. Gaulton, A. *et al.* The ChEMBL database in 2017. *Nucleic Acids Res* **45,** D945–D954 (2017).

2. Wishart, D. S. *et al.* DrugBank: a comprehensive resource for in silico drug discovery and exploration. *Nucleic Acids Res* **34,** D668-72 (2006).

3. Roth, B. L., Lopez, E., Patel, S. & Kroeze, W. K. The multiplicity of serotonin receptors: uselessly diverse molecules or an embarrassment of riches? *Neuroscientist* **6,** 252–262 (2000).

4. Feng, Z. *et al.* Ligand Depot: a data warehouse for ligands bound to macromolecules. *Bioinformatics* **20,** 2153–2155 (2004).

5. Gilson, M. K. *et al.* BindingDB in 2015: A public database for medicinal chemistry, computational chemistry and systems pharmacology. *Nucleic Acids Res* **44,** D1045-53 (2016).

6. Wishart, D. *et al.* T3DB: the toxic exposome database. *Nucleic Acids Res* **43,** D928-34 (2015).

7. Zhu, F. *et al.* Therapeutic target database update 2012: a resource for facilitating target-oriented drug discovery. *Nucleic Acids Res* **40,** D1128-36 (2012).

8. Saubern, S., Guha, R. & Baell, J. B. KNIME Workflow to Assess PAINS Filters in SMARTS Format. Comparison of RDKit and Indigo Cheminformatics Libraries. *Mol Inform* **30,** 847–850 (2011).

9. Berthold, M. R. *et al.* in *Data Analysis, Machine Learning and Applications* (eds.

Preisach, C., Burkhardt, H., Schmidt-Thieme, L. & Decker, R.) 319–326 (Springer Berlin Heidelberg, 2008). doi:10.1007/978-3-540-78246-9_38

10. Hawkins, P. C. D., Skillman, A. G. & Nicholls, A. Comparison of shape-matching and docking as virtual screening tools. *J Med Chem* **50,** 74–82 (2007).

11. OpenEye Scientific Software, Santa Fe, NM. ROCS 3.2.1.4. (2017). http://www.eyesopen.com

12. Hawkins, P. C. D., Skillman, A. G., Warren, G. L., Ellingson, B. A. & Stahl, M. T. Conformer generation with OMEGA: algorithm and validation using high quality structures from the Protein Databank and Cambridge Structural Database. *J Chem Inf Model* **50,** 572–584 (2010).

13. OpenEye Scientific Software, Santa Fe, NM. OMEGA 2.5.1.4. (2017). http://www.eyesopen.com

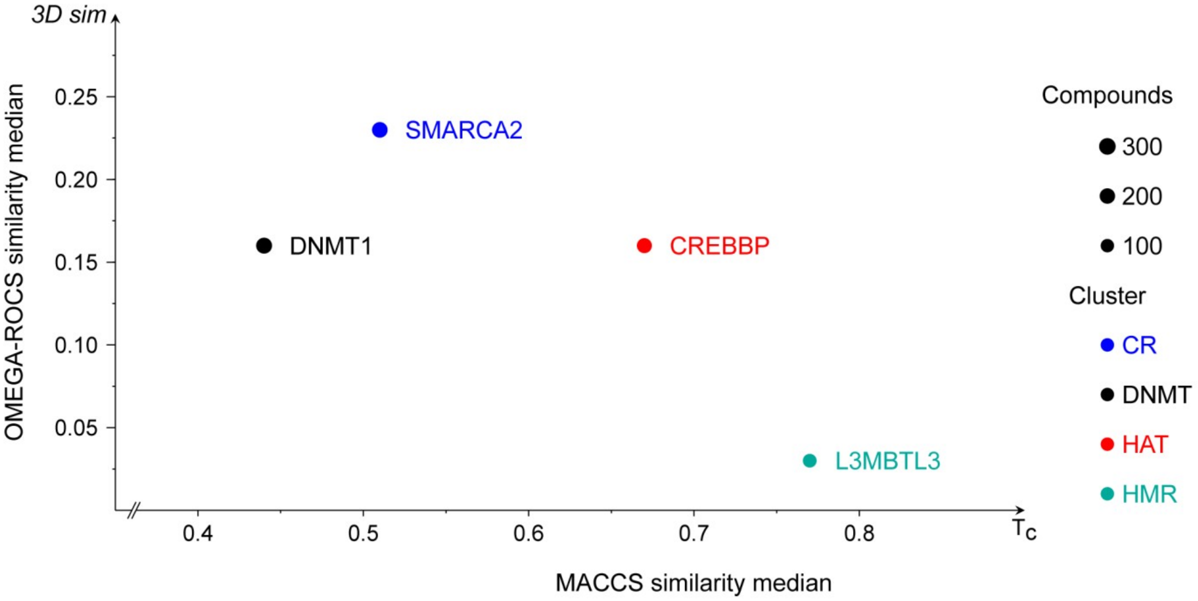

Figure S1. 3D-Consensus Diversity Plot depicting the diversity of the datasets used for the backwards approach. The higher the median similarity either 2D or 3D, the lower diversity of the dataset. The size of the points is proportional to the size of the dataset. The color shows the function of the target. CR: chromatin remodeller; DNMT: DNA-methyltransferase; HAT: histone acetyltransferase; HMR: histone methylation reader.

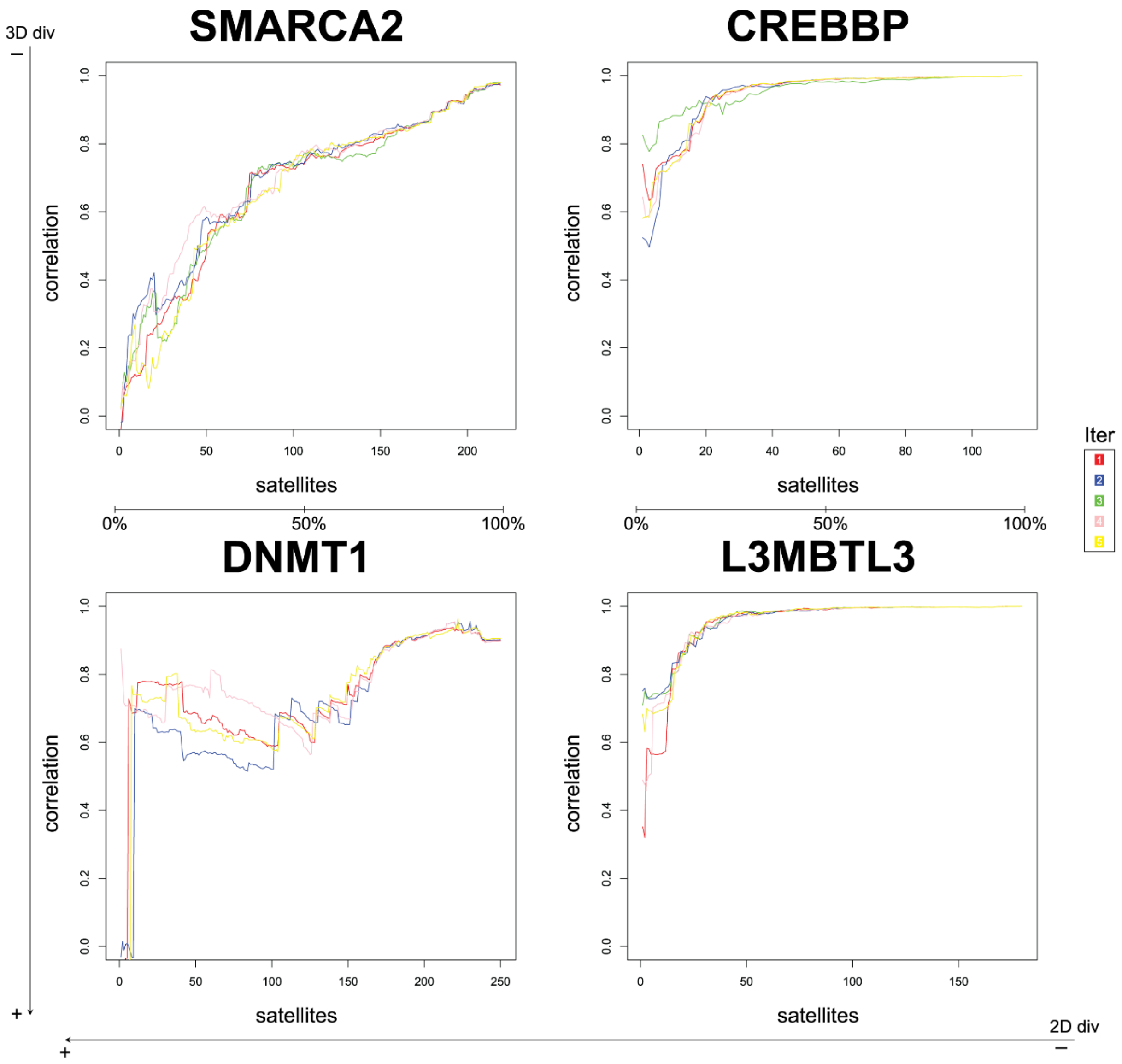

Figure S2. Backwards analysis with 3PCs picking satellites by diversity. The correlation with the results from the whole matrix was calculated with increasing numbers of satellites. Each colored line represents one of the five random sets.

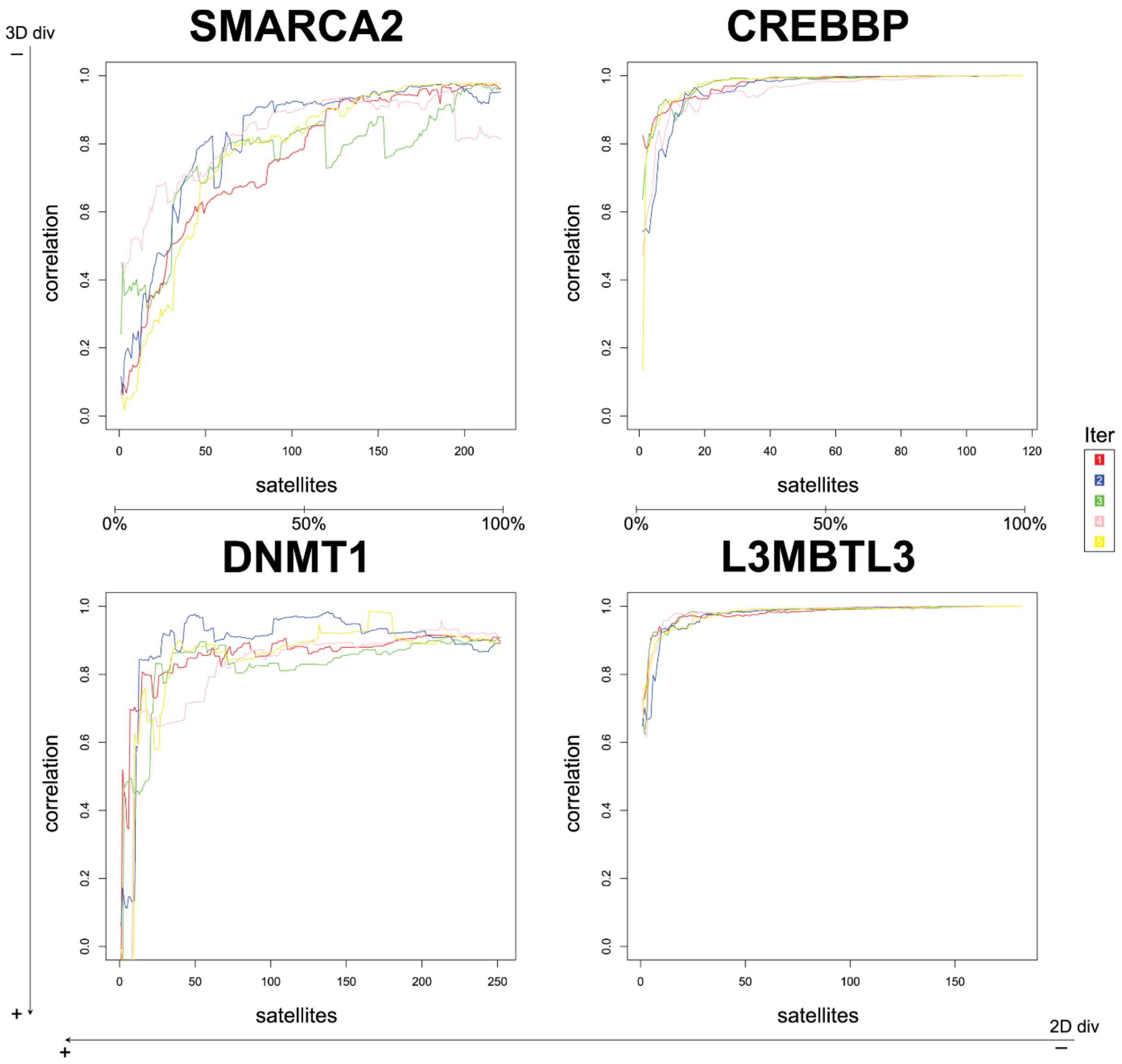

Figure S3. Backwards analysis with 3PCs picking satellites at random. The correlation with the results from the whole matrix was calculated with increasing numbers of satellites. Each colored line represents one of the five random sets.

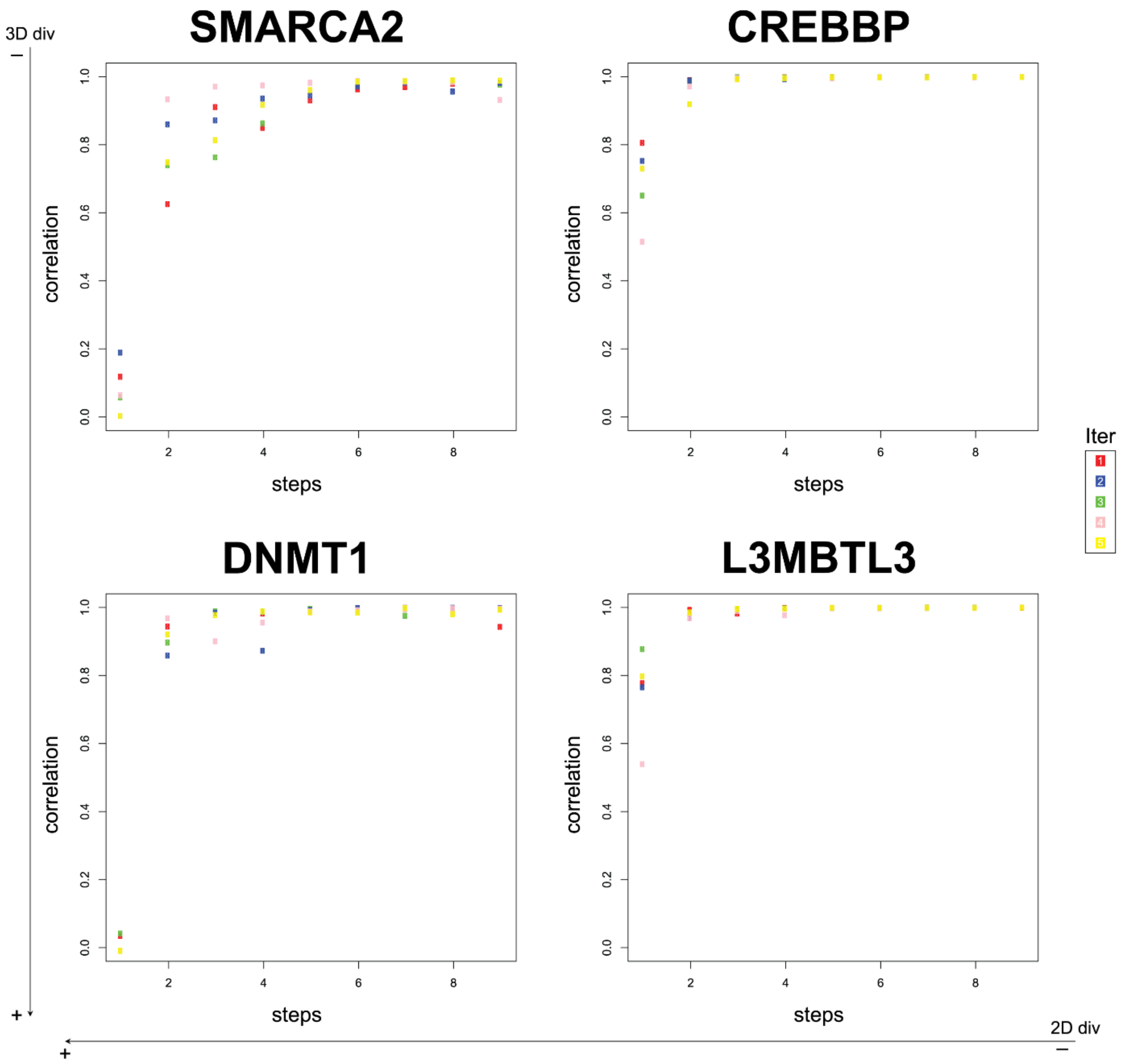

Figure S4. Forward analysis with 2PCs picking satellites at random with step sizes of 10%.

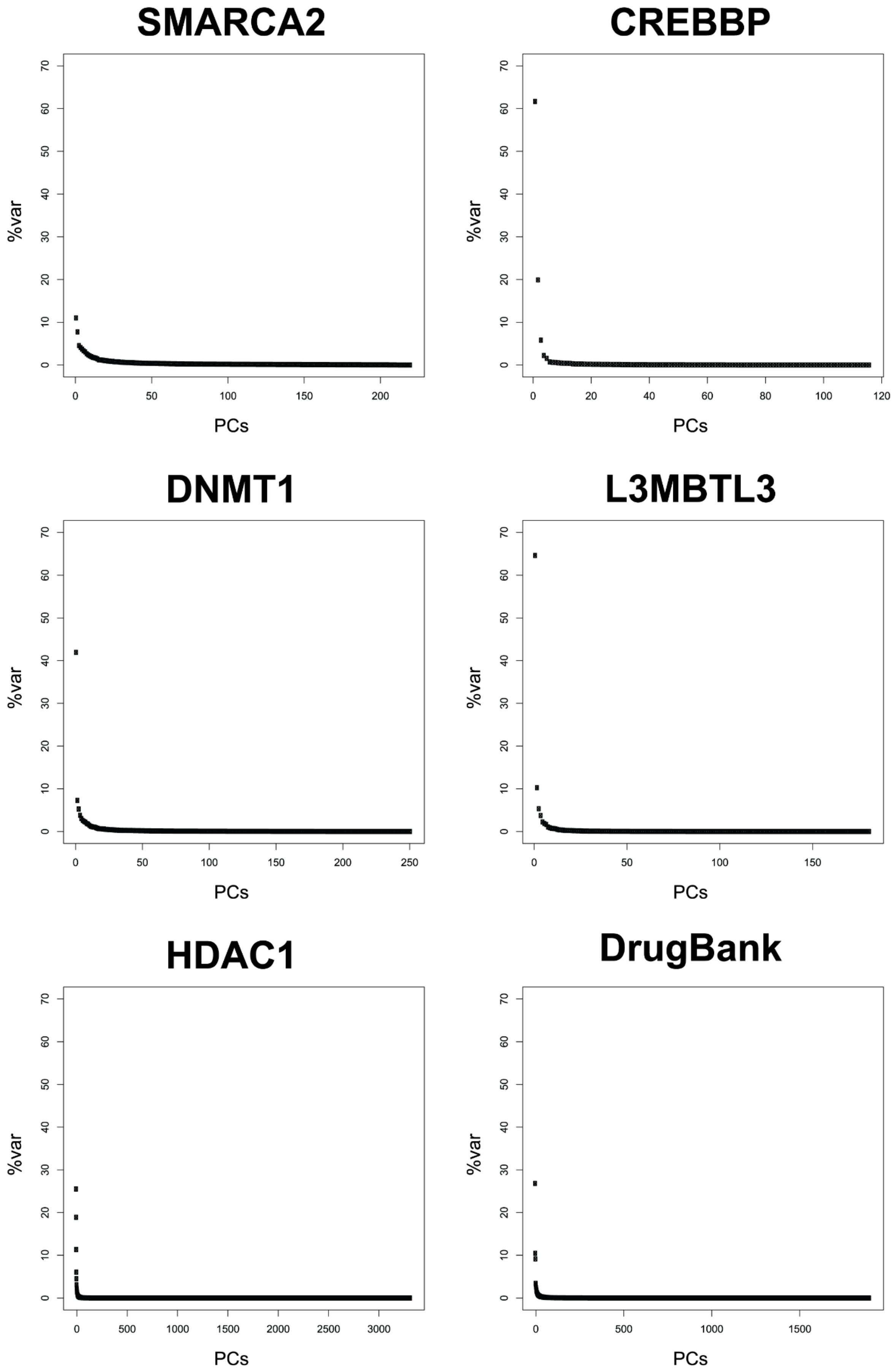

Figure S5. Plot of the percentage of variance explained by each principal component in the studied datasets.
