# Supplementary material for: ChemMaps: Towards an approach for visualizing the chemical space based on adaptive satellite compounds
Source: F1000Res. 2017 Aug 4;6:Chem Inf Sci-1134. Originally published 2017 Jul 17. [Version 2] doi: 10.12688/f1000research.12095.2 (PMC5538041; doi:10.12688/f1000research.12095.2)
Supplement: Peer review history

# Peer review: Referee response for version 2

**DOI:** 10.5256/f1000research.13342.r24805

**Published:** 2017-09-08

**Authors:** Gerald Maggiora

**Affiliations:** 1 BIO5 Institute, University of Arizona, Tucson, AZ, USA

Although the main issue I raised in my earlier review regarding the ‘gold standard’ was not addressed, the authors did make mention of the issue, and they did address some of it in Figure S5. While I’m not totally satisfied with this, I feel that their work is ready for publication, but with the reservation regarding the issue just mentioned. This issue should be considered in more detail in their future work.

 The authors have made some helpful improvements in their manuscript, but there are still some issues they may want to consider to further improve it, although it is not necessary for them to do so.

  

1. Step 1 in the ‘Backwards approach’ is unclear. Perhaps an example would help clarify exactly what is being done. I could not reproduce Step 1 as it is currently written.
2. In step 3 of the ‘Backwards approach’ the authors state that:
  Compute all pair-wise Euclidean distances based on the scores of the 2 or 3 PCs generated in step 2. The set of distances are later used as reference or ‘ *gold standard*’. It should be noted that the “real” distances or true gold standard would consider the whole distance matrix. However, for visualization purposes it is unfeasible to render more than 3 dimensions. Therefore, we selected as reference the best 2D or 3D visualization possible by means of PCA.
  With regard to the underlined text in the authors’ statement above, I believe they are missing the point. The reason for considering the full or a significant subspace for a given compound collection and not the 2- or 3-dimensional subspaces derived from a PCA has nothing to do with the impossibility of rendering the data in more than three dimensions because this ‘gold standard’ is not going to be graphically depicted. The important point is that the distances calculated in this space are exact within the limitations of the methodology and means of data collection used. Hence, they form the best ‘gold standard’ that can be achieved within these limitations. The difficulty with this approach is the need to compute Euclidean distances in the full higher-dimensional space, which may require some additional work. However, this distance matrix need only be computed once. I feel the authors should consider this in the future work. Alternatively, the authors need not necessarily involve the complete space, but they should chose a subspace of sufficient dimension to ensure that a significant percentage of the variance, say greater than 90%, is accounted for as a basis for the gold standard.     
3. In Step 8 the terminology ‘prior steps’ is used. Does this include all prior steps? I think it would be clear to name the actual steps as is done in earlier steps in this section, e.g. step 4 states “Repeat steps 4 and 5 increasing…”
4. Although unnecessary, might the authors comment on less well behave character associated with the iterations of the DNMT1 inhibitors dataset compared to the other datasets depicted in Figure 1.
5. Line 2 in the ‘Forward approach’ should read: “… e.g., step 1 in the backwards approach.”
6. If I understand the %variance plots in Figure S5 correctly, it seems that the %variance for two PCs are for three of the databases greater than or equal to 50%, but three are less than 50%, with the SMARCA2 database only reaching about 20%. Is the %variance in the latter three cases, but especially for the SMARCA2 database, sufficient to provide a basis for a reasonably faithful representation for all of the respective datasets?

I have read this submission. I believe that I have an appropriate level of expertise to confirm that it is of an acceptable scientific standard, however I have significant reservations, as outlined above.

# Peer review: Referee response for version 2

**DOI:** 10.5256/f1000research.13342.r24807

**Published:** 2017-08-14

**Authors:** Dmitry I. Osolodkin

**Affiliations:** 1 Institute of Poliomyelitis and Viral Encephalitides, Chumakov FSC R&D IBP RAS, Moscow, Russian Federation; 2 Department of Chemistry, Lomonosov Moscow State University, Moscow, Russian Federation

I thank the authors for addressing most of the issues. However, two problems still appear.

1. The authors say that "selecting 2 PCs the performance is slightly better and more stable (compare Figure 1 and Figure 2 against Figure S2 and Figure S3)" than with 3 PCs. When I compare Figure 1 with Figure S2, it seems to me that 3 PCs are at least not worse than 2 PCs, and maybe even slightly better on the basis of smaller difference between runs. Thus, without a quantitative measure it cannot be said that performance of 2 PCs is better, it may be only said that 3 PCs do not provide improvement over 2 PCs (though it is not obvious from the plots), and if 3 PCs scheme has higher computational demands, it may also be mentioned.
2. The compound standardisation procedure (Supplementary Methods) looks as it is taken from a different manuscript. For example, compound activity is mentioned, although not used in this study. It is also not stated that standardisation procedure was not applied to DrugBank (at least to the SDF file available in the supplementary dataset).

I have read this submission. I believe that I have an appropriate level of expertise to confirm that it is of an acceptable scientific standard, however I have significant reservations, as outlined above.

# Peer review: Referee response for version 2

**DOI:** 10.5256/f1000research.13342.r24806

**Published:** 2017-08-07

**Authors:** Jean-Louis Reymond

**Affiliations:** 1 Department of Chemistry and Biochemistry, University of Bern, Bern, Switzerland

Thanks for citing our paper on similarity maps. However the key point is missing: in our 2015 paper (Awale & Reymond 2015) we computed similarity values for all ChEMBL molecules (> 1 million) to only 100 molecules used as reference (the same as "satellites" here), and not the full similarity matrix. We then performed a PCA of the resulting similarity fingerprint to obtain a 2D-map (see the interactive "similarity-mapplets" web portal at www.gdb.unibe.ch). We have also published another implementation of similarity maps to visuzalize the Protein DataBank, using the same principle of choosing a limited set of sattelites only (Jin *et al. *2015). 

 The authors are doing exactly the same similarity calculation here using a limited set of reference compounds, as we did then, nothing is new. What is interesting in the present report is to look at how the selected set of "satellites" influences the PCA-map.

I have read this submission. I believe that I have an appropriate level of expertise to confirm that it is of an acceptable scientific standard, however I have significant reservations, as outlined above.

## References

[1] AwaleMReymondJL: Similarity Mapplet: Interactive Visualization of the Directory of Useful Decoys and ChEMBL in High Dimensional Chemical Spaces. *J Chem Inf Model*.2015;55(8) : 10.1021/acs.jcim.5b00182 1509-16 10.1021/acs.jcim.5b00182 26207526

[2] JinXAwaleMZassoMKostroDPatinyLReymondJL: PDB-Explorer: a web-based interactive map of the protein data bank in shape space. *BMC Bioinformatics*.2015;16: 10.1186/s12859-015-0776-9 339 10.1186/s12859-015-0776-9 26493835PMC4619230

# Response

**Published:** 2017-08-08

**Authors:** José L. Medina-Franco

**Affiliations:** Universidad Nacional Autónoma de México, Mexico

Dear Dr. Reymond, thank you for your feedback. Following your comment in our initial submission (point 1 in your report), we have acknowledged your approach. Indeed, ChemMaps is highly related to your work (as well as to Chem-GPS). A key difference, however, ChemMaps is meant to analyze individual data sets of compounds that might be included or not in ChEMBL or Protein Data Bank. This would be useful for corporate databases. We are not proposing a defined set of standard compounds or satellites. The set of satellites would be "dynamic" and dependent on the data sets.

# Peer review: Referee response for version 1

**DOI:** 10.5256/f1000research.13086.r24277

**Published:** 2017-07-31

**Authors:** Jean-Louis Reymond

**Affiliations:** 1 Department of Chemistry and Biochemistry, University of Bern, Bern, Switzerland

J. Jesús Naveja *et al* present a methodology for representation of chemical space of small sets of compounds. In general, the approach involves selection of satellite compounds from the database, computing the similarities of all compounds in the database to these satellites, and finally projection of the resulting similarity matrix using principal component analysis. J. Jesús Naveja *et al* further report various methods for selecting satellite compounds (backward or forward selection approach; selection at random or selection by diversity check) and show how the number of selected satellite compounds influence the quality of projection.

 Comments:

1. The authors are completely hiding the fact that similarity mapping is quite well-known and absolutely not new, the authors should read and cite Awale et al., J. Chem. Inf. Model., 2015, 55 (8), pp 1509–1516 and the detailed discussion of literature precedents on similarity mapping presented therein.
2. The authors compare their satellites to the satellite compounds used by T. Oprea in his 2001 approach to mapping chemical space. Obviously either they did not read Oprea’s paper or they misunderstood it: Oprea’s satellites are artificial molecules with extreme properties such as to orient the PCA projection and stretch its dimensions in reproducible directions. However the projection is simply PCA, and does not involve similarity mapping. In similarity mapping the satellites are molecules from within the database to which similarities are calculated.
3. In the abstract, author mentioned that “3D diversity played a secondary role, although it becomes increasingly relevant as 2D diversity increases”. However, I didn't found the relevant explanation in main text supporting this statement.
4. Figure 1 and Figure 2: The five random sets in the legend. Its not clear exactly what the author meant by five random sets. As per my understanding the author used the complete set of compounds for each target and what is changing is the random selection of satellites, which is repeated for five times.
5. In case of forward selection approach: “..With that in mind, we decided to design a method that starts with a given percentage of the database as satellites, and then keeps adding a proportion of them until the correlation between the former and the updated data is of at least 0.9. ” The correlation between projections obtained from the current set of satellites and projections obtained from former set of satellites might well be high, but still the correlation to the projection obtained from the complete similarity matrix is low. How one can assure the quality of projection in this case?
6. For all plots axis labels are too small to read.

I have read this submission. I believe that I have an appropriate level of expertise to confirm that it is of an acceptable scientific standard.

## References

[1] AwaleMReymondJL: Similarity Mapplet: Interactive Visualization of the Directory of Useful Decoys and ChEMBL in High Dimensional Chemical Spaces. *J Chem Inf Model*.2015;55(8) : 10.1021/acs.jcim.5b00182 1509-16 10.1021/acs.jcim.5b00182 26207526

# Response

**Published:** 2017-08-02

**Authors:** José L. Medina-Franco

**Affiliations:** Universidad Nacional Autónoma de México, Mexico

Dear Dr. Reymond, thank you for your comments to this Research Note.

Regarding the modifications we have done considering your comments:

- In the Introduction we briefly discuss other similarity approaches to visualize the chemical space. We have expanded that discussion there with a reference to the Similarity Mapplet approach.
- We did not intend to imply that Oprea’s and Gottfires’ ChemGPS approach is based on structural similarity. To clarify this point we rephrased that in the introduction.
- In the corresponding Figures legends we changed “random sets” with “iterations”.
- We added to the Supplementary Information a discussion on the correlation of the complete similarity matrix Euclidean distances and using only 2 and 3PCs. However, we would like to highlight that our approach is intended to approximate the best possible chemical space visualization using PCA. This last is given by the first 3PCs at most.
- We augmented the font size in all figures.

# Peer review: Referee response for version 1

**DOI:** 10.5256/f1000research.13086.r24276

**Published:** 2017-07-28

**Authors:** Dmitry I. Osolodkin

**Affiliations:** 1 Institute of Poliomyelitis and Viral Encephalitides, Chumakov FSC R&D IBP RAS, Moscow, Russian Federation; 2 Department of Chemistry, Lomonosov Moscow State University, Moscow, Russian Federation

The paper under consideration presents an elegant approach to efficient mapping of chemical space using principal component analysis. Being technically sound in general, well-written and easily understandable, the paper lacks several technical details without which it is not complete. In particular:

  

1. The concept of 'chemical satellites' is discussed in a rather concise manner, a bit more details may be added and the seminal paper by Oprea & Gottfries [1] needs to be cited. The approach suggested here is rather different from the Oprea's one, because satellites are defined there as intentional outliers, whereas in the current work they are just extracted from the mapped dataset. This difference should be stated in a clearer way.
2. Dataset processing routine is not presented. Although the suggested technique would work on totally random datasets (by the way, addition of such a dataset to the list of examples would be beneficial and illustrative), standardization of structures should be performed for consistency and for more informative application of similarity measures. Targeted datasets in the supplement look standardized, but DrugBank contains metal ions, unconnected molecules, and macromolecules, all of which may significantly distort the comparison. For HDAC1 inhibitors the procedure to obtain this dataset from ChEMBL should be provided, because simple target keyword search for 'hdac1' gives 9 different datasets.
3. Diversity of datasets may be additionally illustrated by any of currently available visualization methods. A method that clearly shows compound clustering or diversity of the dataset would be preferred.
4. Visual comparison of figures is not sufficient to make conclusions about preference of random selection over diversity-based (Figures 1, 2, S2, S3). Differences are visible, but their importance and significance are not obvious (maybe just for me), so use of a quantitative measure would be highly appreciated. Random selection shows sometimes lower stability of the backwards analysis (larger difference between the iterations), and this observation could be discussed.
5. Some analysis of the technique applicability domain would significantly improve the conclusions of the paper. One parameter that deserves attention is dataset diversity threshold above which the technique becomes unstable or less useful. Will it work good for totally random or intentionally diverse compounds or for datasets with two or three large congeneric series? A slightly more thorough characterization of example datasets would be useful to deal with this question.

I have read this submission. I believe that I have an appropriate level of expertise to confirm that it is of an acceptable scientific standard, however I have significant reservations, as outlined above.

## References

[1] OpreaTGottfriesJ: Chemography: The Art of Navigating in Chemical Space. *Journal of Combinatorial Chemistry*.2001;3(2) : 10.1021/cc0000388 157-166 10.1021/cc0000388 11300855

# Response

**Published:** 2017-08-02

**Authors:** José L. Medina-Franco

**Affiliations:** Universidad Nacional Autónoma de México, Mexico

Dear Dr Osolodkin, thank you, we highly appreciate your comments to this Research Note.

Regarding the modifications we have done considering your comments:

- We added a citation to the first publication related to ChemGPS by Oprea and Gottfries. In the Introduction, we further, although briefly (given the extension limit of a Research Note), explained the differences among these two approaches.
- We added a Supplementary Information file describing the data curation methodology used. -We also added the HDAC1 dataset to the supplementary files.
- Supplementary Figure 1 should address the visualization of the diversity of the datasets.
- We find quite interesting your observation about quantifying the stability of the iterations, as well as that about determining the applicability domain of the approach (including defining a diversity threshold). Based on this Research Note we are planning an extensive study fully addressing these concerns.

# Peer review: Referee response for version 1

**DOI:** 10.5256/f1000research.13086.r24274

**Published:** 2017-07-20

**Authors:** Gerald Maggiora

**Affiliations:** 1 BIO5 Institute, University of Arizona, Tucson, AZ, USA

Graphically representing coordinate-based chemical spaces requires some type of dimensionality reduction. One method involves the use of similarity matrices treated as data matrices that are subsequently subjected to principal component analysis (PCA). The first two or three PCs are then used as a basis to graphically depict the chemical space. Although this approach works reasonably well, the size of chemical spaces that can be treated is somewhat limited, since the PCA transformation requires diagonalizing a matrix whose dimension is equal to the number of molecules in the chemical space of interest. The work of Naveja and Medina-Franco seeks to overcome this limitation by building a lower dimensional representation of chemical space in a stepwise manner using “backwards” or “forward” procedures. While the method has the potential for accomplishing their goals, it does not in my estimation provide a sufficiently rigorous test of the approximations that are the foundation of their approach. For this reason additional work needs to be done before their method can be applied with confidence.

 My objection is based on the authors’ use of the first 2 or 3 PCs as the ‘gold standard’ for representing of the entire chemical space, and as a basis for all subsequent comparisons of the approximate chemical spaces. I would at least like to see what percent of the total sample variance is accounted for by these PCs. If it is an insignificant amount, then approximating these PCs by whatever method will not produce a sufficiently accurate model of the chemical space and their model will have to be improved. The true ‘gold standard’ is the original set of column vectors in their data matrix from which the PCs are obtained. This will produce the ‘true’ distance between ‘molecular points’ in the full dimensional chemical space, but because of its very high dimension computing distances in the original chemical space can be a problem. An alternative is to carry out the PCA and choose a larger subset of PCs (say 6 or 8) that do account for most of the sample variance and then use these in the correlation or error analysis.

I have read this submission. I believe that I have an appropriate level of expertise to confirm that it is of an acceptable scientific standard, however I have significant reservations, as outlined above.

# Response

**Published:** 2017-07-27

**Authors:** José L. Medina-Franco

**Affiliations:** Universidad Nacional Autónoma de México, Mexico

Dear Dr. Maggiora,

 We thank you for your feedback on this Application Note. We entirely agree with your comment that if the variance captured by the first 2 or 3 PCs is not high enough, the visual representation of the chemical space will not be meaningful. For the data sets included in this work, we have seen that the variance is high. We also agree that formally speaking the ´true gold standard´ would involve computing the distances for the full matrix. Based on your feedback we are preparing a revised version of this manuscript.
